# Test characteristics of point-of-care ultrasonography in patients with acute kidney injury

**DOI:** 10.1186/s13089-023-00352-3

**Published:** 2024-02-22

**Authors:** Mathilde Gaudreau-Simard, Tana Saiyin, Matthew D. F. Mcinnes, Sydney Ruller, Edward G. Clark, Krista Wooller, Elaine Kilabuk, Alan J. Forster, Michael Y. Woo

**Affiliations:** 1 Division of General Internal Medicine, The Ottawa Hospital-General Campus, 501 Smyth Road, Ottawa, ON K1H 8L6 Canada; 2 Ottawa Hospital Research Institute, Ottawa, ON Canada; 3 Department of Medicine, University of Ottawa, Ottawa, ON Canada; 4 Department of Diagnostic Imaging, University of Ottawa, Ottawa, ON Canada; 5 Division of Nephrology, The Ottawa Hospital, Ottawa, ON Canada; 6 Department of Emergency Medicine, University of Ottawa, Ottawa, ON Canada

**Keywords:** Point-of-care ultrasound, QI, PoCUS, AKI, Diagnostic accuracy

## Abstract

**Background:**

Acute kidney injury is a common disorder that is associated with significant morbidity and mortality. Point-of-care ultrasonography (PoCUS) is an imaging modality performed at the bedside and is used to assess for obstructive causes of acute kidney injury. Little is known about the test characteristics of PoCUS in patients with acute kidney injury.

**Objective:**

Our primary objective was to describe the test characteristics of PoCUS for the detection of hydronephrosis in patients presenting with acute kidney injury at our centre. Our secondary objective was to describe the current rate of use of PoCUS for this indication.

**Results:**

In total, 7873 patients were identified between June 1, 2019 and April 30, 2021, with 4611 meeting inclusion criteria. Of these, 94 patients (2%) underwent PoCUS, and 65 patients underwent both PoCUS and reference standard, for a total of 124 kidneys included in our diagnostic accuracy analysis. The prevalence of hydronephrosis in our cohort was 33% (95% CI 25–41%). PoCUS had a sensitivity of 85% (95% CI 71–94%) and specificity of 78% (95% CI 68–87%) for the detection of hydronephrosis.

**Conclusion:**

We describe the test characteristics of PoCUS for the detection of hydronephrosis in a cohort of patients with acute kidney injury. The low uptake of this test presents an opportunity for quality improvement work to increase its use for this indication.

**Supplementary Information:**

The online version contains supplementary material available at 10.1186/s13089-023-00352-3.

## Background

Acute kidney injury is a common disorder and is associated with significant morbidity and mortality [1, 2]. Studies have shown that 5–10% of patients admitted to hospital have acute kidney injury at the time of their initial presentation [3, 4]. Post-renal acute kidney injury, resulting from obstruction of urinary flow, is responsible for 5–10% of these cases [4–6]. Considering its reversibility [3], it is an important diagnosis not to miss or delay. Post-renal acute kidney injury cannot be identified by physical examination alone, and as such, imaging is required to secure the diagnosis [7]. Ultrasonography is the test of choice for identifying hydronephrosis, the cardinal sign of post-renal acute kidney injury [8, 9].

Point-of-care ultrasonography (PoCUS) is performed at the bedside and interpreted in real-time by the treating physician. The relative advantage of PoCUS lies in its portability, speed and availability as compared to diagnostic imaging performed in the radiology department [10]. As such, it has great potential for the evaluation of patients with acute kidney injury [11]. In patients presenting with renal colic, kidney PoCUS has been shown to have a sensitivity of 73–92% and a specificity of 59–83% for the detection of hydronephrosis [12–21]. This evidence has been extrapolated to patients with acute kidney injury and several medical societies have made kidney PoCUS a core competency for their specialty [22–24]. However, the test characteristics of kidney PoCUS in patients presenting with acute kidney injury is not well described in the literature [25, 26], which may limit its widespread uptake.

### Objective

Our primary objective was to describe the test characteristics of PoCUS in patients presenting with AKI at our centre. Our secondary objective was to describe the current rate of use of PoCUS for this indication, at our centre.

## Methods

### Study design

This is a retrospective cohort study of adults presenting to the emergency department (ED) of the Ottawa Hospital with acute kidney injury who underwent kidney PoCUS, between June 1, 2019, and April 30, 2021. This is part of a larger quality improvement project aimed at increasing the uptake of kidney PoCUS in this patient population.

### Participants

Through our data warehouse, we used ICD-10 coding to identify adults presenting to the ED of the Ottawa Hospital with acute kidney injury, between June 1, 2019, and April 30, 2021. The Ottawa Hospital is a 1335-bed academic tertiary care centre with over 160,000 ED patient-visits per year. We excluded patients who were dialysis-dependent, prior kidney transplant recipients and those who did not meet the Kidney Disease Improving Global Outcomes criteria for stage 1 acute kidney injury (≥ 26.5 umol/L or 1.5 × increase from baseline serum creatinine) [27]. If no previous creatinine was available, patients were included if their creatinine at presentation was ≥ 26.5 μmol/L above the upper limit of normal for their sex (ULN) (ULN is 84 μmol/L for women and 100 μmol/L for men). Finally, we excluded patients who were discharged directly from the ED.

Within our cohort, we identified patients who had undergone kidney PoCUS on presentation. First, encounter notes containing one of 15 keywords synonymous with PoCUS^1^ were identified and patients were included if the PoCUS included the kidney(s). Second, all patient Medical Record Numbers were manually entered into our imaging archiving software QpathE (Telexy Healthcare, Maple Ridge, BC, Canada) to identify exams that may have been missed through our first method. A PoCUS was considered positive if the presence of hydronephrosis was recorded either in the physician note or in the QpathE reporting worksheet. A test was considered negative if the absence of hydronephrosis was recorded in either of these mediums. If a PoCUS scan was archived but no interpretation was documented, the patient was excluded. A PoCUS was considered indeterminate if it was reported as inconclusive or not interpretable. Indeterminate scans were excluded from our diagnostic accuracy analysis. The reasoning behind this consensus decision is that, at our centre, PoCUS providers are taught to fall back on their history and physical examination for clinical decision-making when they obtain an indeterminate scan. This approach is analogous to no PoCUS having been performed and justifies the exclusion of indeterminate tests from our analysis. This process was performed by four independent reviewers. If there was uncertainty about whether a patient should be included, the encounter was reviewed, and a decision was made by the project lead (MGS).

We performed a health records review of our final patient population. We recorded age, sex, baseline and creatinine on presentation, comorbidities by Charleston index, ED diagnosis, admission service, PoCUS date and time If the PoCUS scan was not archived in QpathE, the time of the scan was defined as the time of exam recorded in the encounter note or the time of the physician's initial assessment if the former was unavailable. The PoCUS provider and their credentials were recorded. Emergency physicians were considered credentialed if they completed an introductory PoCUS course, obtained at least 50 supervised or reviewed scans and successfully completed an examination. There was no credentialing process in place for other subspecialities at the time of this review. Finally, a PoCUS expert reviewed all PoCUS scans that had been archived and provided an interpretation (MYW).

We then identified whether patients underwent radiology-performed ultrasound (RADUS) or computed tomography (CT) within 48 h of PoCUS. If radiology-performed imaging was performed first (prior to PoCUS), the patient was excluded. We recorded indication, time of imaging, and imaging result. For patients who underwent both reference standard and index test, we recorded whether Foley was inserted on presentation.

For patients who underwent PoCUS but did not undergo reference standard, a chart review was used to identify if obstructive uropathy had been missed. We identified if imaging was done later (> 48 h) in the index admission, recorded creatinine on hospital discharge, creatinine on post-hospital follow-up, and reviewed clinical notes to determine if an alternate cause of AKI was identified.

### Test methods

#### Index test

The index test was PoCUS of the kidneys, performed by the treating physician. All exams were performed using the Philips Sparq or the Fujifilm SonoSite X-Porte.

#### Reference standard

The reference standard was computed tomography or radiology ultrasound performed within 48 h after PoCUS. If both computed tomography and radiology-performed ultrasound were performed, the reference standard was computed tomography.

### Statistical analysis

Summary statistics (sum, mean, and median) were generated using Excel. We performed a data distribution analysis to inform choice of the statistic to report for creatinine and time to imaging. Diagnostic accuracy and 95% confidence intervals were determined using the EpiR package in R statistical software. Sensitivity analysis was performed after exclusion of all patients having had a Foley catheter inserted in the ED.

### Ethical considerations

This study was part of a larger quality improvement project aimed at increasing the use of PoCUS in patients with acute kidney injury. We obtained an exemption from the Ottawa Health Science Network Research Ethics Board and registered our project in the IQ@TOH Project Registry prior to the project start.

## Results

### Participants

**Fig. 1:**
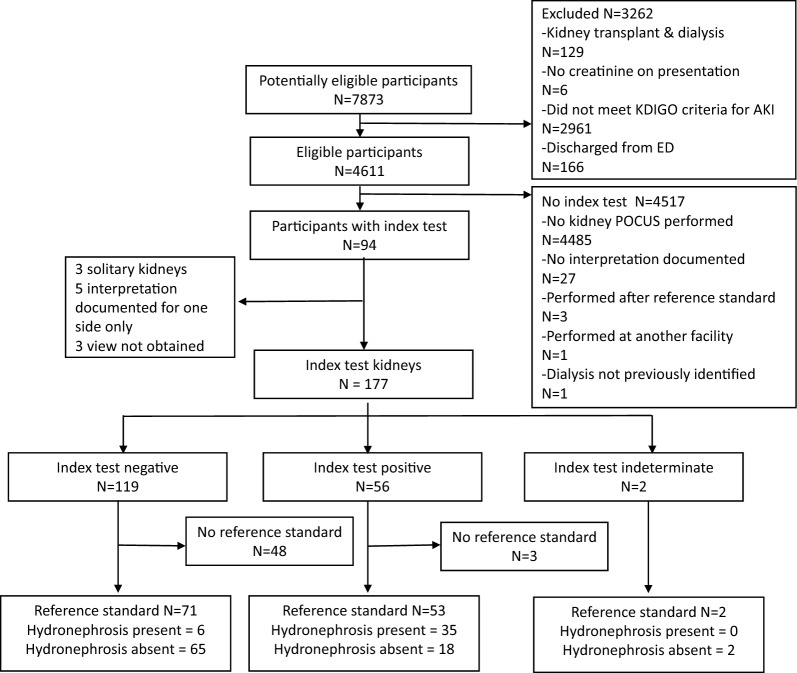
STARD participant

In total, 7873 patients were identified and 4611 met the inclusion criteria. Of these, 94 (2%) patients underwent the index test, for a total of 177 kidney scans performed, representing our cohort. Of these, 65 patients had the reference standard, totaling 126 index tests, 2 index scans were indeterminate and excluded. In the end, 124 tests were included in our diagnostic accuracy analysis. The remaining 53 kidney scans (27 patients) did not undergo a reference standard and were subject to further chart review (Fig. 1).

The mean age of our cohort was 72 years (SD 15), with 66% of participants being male (Table 1). The three most frequent ED primary diagnoses were acute kidney injury (35%), urinary tract infection (14%), and sepsis (13%). PoCUS was performed by 44 different providers with the majority being credentialed emergency medicine (EM) residents (39%) and attendings (34%). The remaining providers were non-credentialed EM trainees (9%) and subspecialty residents (18%).

**Table 1:** Cohort characteristics

	*N* = 94
Age in years, mean (SD)	72 (15)
Sex, *N* (%)	
Male	64 (66%)
Baseline creatinine (umol/L) [*N* = 72], median (IQR)	107 (84–150)
Creatinine on presentation (umol/L) [*N* = 94], median (IQR)	275 (197–482)
Comorbidities (Charlson index), *N* (%)	
Diabetes mellitus	382 (40%)
Non-metastatic malignancy	21 (22%)
Renal disease	20 (21%)
Liver disease	6 (6%)
Congestive heart failure	11 (12%)
Metastatic malignancy	5 (5%)
ED primary diagnosis, *N* (%)	
Acute kidney injury	33 (35%)
Urinary tract infection	13 (14%)
Sepsis NYD	12 (13%)
Congestive heart failure	5 (5%)
Pneumonia	3 (3%)
Delirium/AMS	3 (3%)
Nephrolithiasis	3 (3%)
Ureteric obstruction	1 (1%)
Prostate mass	1 (1%)
Other	20 (21%)
Admission service *N* (%)	
Internal or family medicine	55 (58%)
Nephrology	13 (14%)
Urology	12 (13%)
Critical care	4 (4%)
Other	10 (11%)
POCUS user specialty and credentials *N* (%)	*N* = 44
EM attending, credentialed	15 (34%)
EM resident, credentialed	17 (39%)
EM resident, non-credentialed	4 (9%)
Consultant, non-credentialed	8 (18%)
Reference standard [*N* = 65]	
CT	44 (67%)
RADUS	21 (33%)
Time from POCUS to reference standard [*N* = 65]	
CT, median (IQR)	2 h 54 m (1 h 44 m–9 h 38 m)
RADUS, median (IQR)	11 h 38 m (9 h 00 m–24 h 45 m)
Foley insertion [*N* = 65]	31 (48%)

### Test results

A total of 124 kidneys were imaged using both the index test and the reference standard (Table 2). The prevalence of hydronephrosis was 33% (95% CI 25–42%). We found that PoCUS had a sensitivity of 85% (95% CI 71–94%) and specificity of 78% (95% CI 68–87%) for the detection of hydronephrosis in our cohort. In addition, PoCUS had a positive likelihood ratio (LR+) of 3.94 (95% CI 2.57–6.04) and a negative likelihood ratio (LR-) of 0.19 (95% CI 0.09 to 0.39) for the detection of hydronephrosis in our cohort.

**Table 2:** Test characteristics of PoCUS in detecting hydronephrosis

	CT or RADUS shows any hydronephrosis	CT or RADUS shows no hydronephrosis	Total
POCUS shows any hydronephrosis	TP = 35	FP = 18	53
POCUS shows no hydronephrosis	FN = 6	TN = 65	71
Total	41	83	124

### False negatives

We found a total of 6 false negative PoCUS results, representing 5 patients. Reference standard graded the hydronephrosis in these kidneys as mild (*N* = 2), moderate (*N* = 3) and moderate to severe (*N* = 1) (Additional file 1: Table S1). Five of these false negatives (representing 4 patients) were archived. PoCUS expert review confirmed that hydronephrosis was present, though image quality was noted to be poor in all. Chart review identified that 4/5 patients were male, and past medical history was significant for urological cancer (2 patients), abdominal cancer (1 patient) and recurrent UTI (1 patient). ED primary diagnosis was acute kidney injury in 3/5 cases (Additional file 1: Table S2).

### False positives and sensitivity analysis

**Fig. 2:**
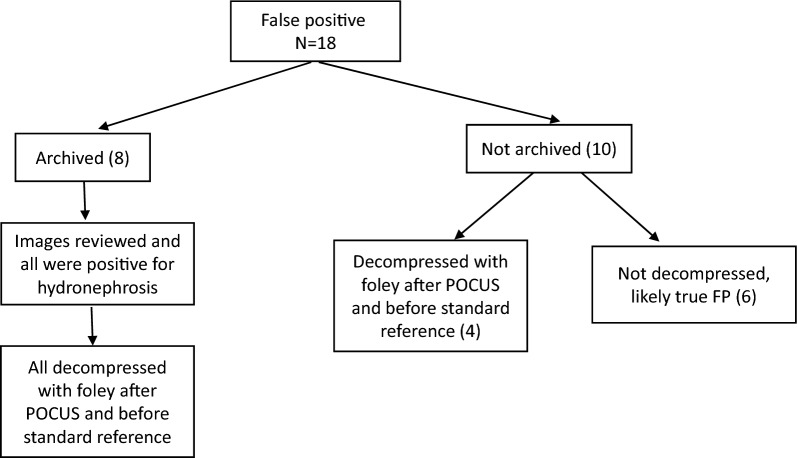
False positive POCUS

We report 18 false positive PoCUS results, which represents 13 patients in our cohort. Eight scans were achieved, and all were noted to be positive for hydronephrosis upon expert review (Fig. 2). Ten were not archived, and of these, 4 underwent decompression with foley.

Considering that decompression with Foley catheter may have led to a discrepancy between index test and reference standard, we performed a second diagnostic accuracy analysis after excluding patients who had a Foley inserted in the ED. In this subgroup, we found that for the detection of hydronephrosis, PoCUS had a sensitivity of 80% (95% CI 59–93%), a specificity of 89% (95% CI 75–97%), a LR+ of 7.40 (95% CI 2.87–19.06) and a LR− of 0.22 (95% CI 0.10–0.50) (Table 3).

**Table 3:** Sensitivity analysis after removing all patients who had a Foley inserted

	CT or RADUS shows any hydronephrosis	CT or RADUS shows no hydronephrosis	Total
POCUS shows any hydronephrosis	TP = 20	FP = 4	24
POCUS shows no hydronephrosis	FN = 5	TN = 33	38
Total	25	37	62

### PoCUS with no reference standard

**Fig. 3:**
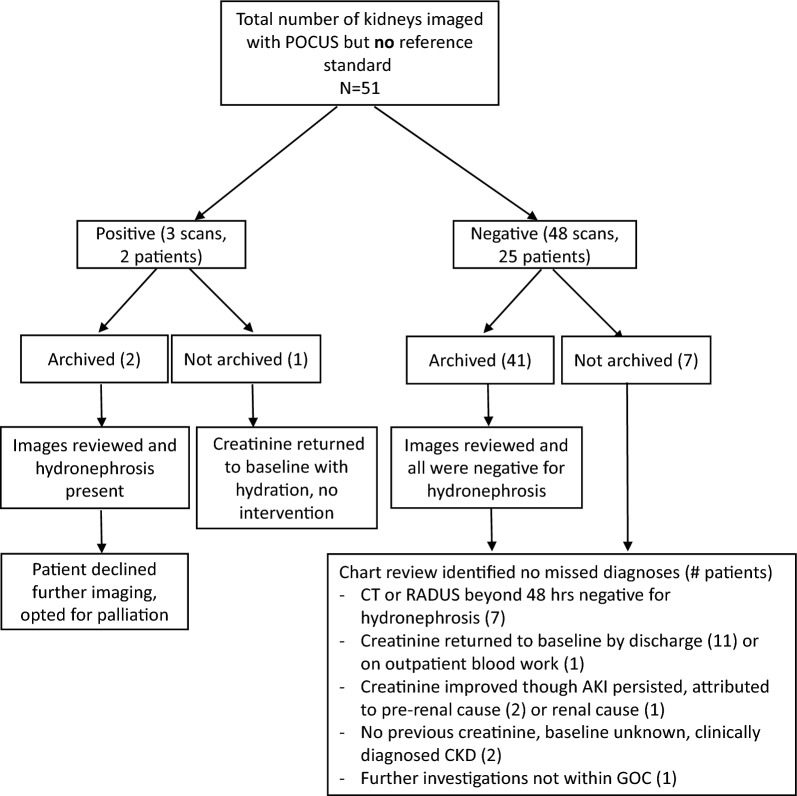
POCUS without the reference standard

A total of 53 PoCUS scans (27 patients) were done without reference standard and therefore were not included in our diagnostic accuracy analysis. We conducted an image review of archived scans and chart review for all cases and identified no missed diagnoses of obstructive uropathy in this subgroup (Fig. 3).

## Discussion

We describe the use of PoCUS in our cohort of patients presenting with AKI. For patients who underwent the reference standard, we report a sensitivity of 85%, a specificity of 78%, a LR+ of 3.94 and a LR− of 0.19. For patients who did not undergo the reference standard, we report no missed diagnoses via chart review. Our review reveals that PoCUS is infrequently used in this patient population at our centre.

Based on our review, PoCUS may be considered in the assessment of patients presenting to the ED with acute kidney injury. When incorporating PoCUS findings into clinical decision-making, clinicians must apply Bayesian reasoning and consider the patient’s pretest probability of obstructive uropathy as the cause of their acute kidney injury.

Our results are comparable to the reported tests characteristics of kidney PoCUS in patients presenting with renal colic. In this population, Herbst et al. [12] found that kidney PoCUS has a sensitivity of 72.6% and a specificity of 73.3% for the detection of hydronephrosis using computed tomography as the reference standard, whereas Pathan et al. report a sensitivity of 81.1% and a specificity of 59.4% for kidney PoCUS with computed tomography as the reference standard. Using computed tomography or radiology-performed ultrasound as the reference standard, Sibley et al. [18] report that kidney PoCUS has a sensitivity of 77.1% and specificity of 71.8% for the detection of hydronephrosis in patients presenting with suspected renal colic [17]. Our study expands on this body of evidence by describing test characteristics of kidney PoCUS in a cohort of patients presenting with acute kidney injury. The higher sensitivity and specificity reported by our study reflects differences in reference standard and target population.

In our study, the reference standard was computed tomography in 67% of cases. While computed tomography is the standard of care to assess for nephrolithiasis in patients presenting with renal colic [28], ultrasound is the test of choice for the evaluation of post-renal acute kidney injury [8, 9]. As such, diagnostic accuracy studies of PoCUS in renal colic have largely used computed tomography as the reference standard, whereas our study included a higher proportion of radiology-performed ultrasound. In the studies by Herbst and Pathan, the reference standard was solely computed tomography, and in Sibley et al., the reference standard was computed tomography in 85% of cases [12, 17, 18]. Considering that both radiology and point-of-care ultrasound are known to have lower sensitivity and specificity than computed tomography for detecting hydronephrosis, these differences were likely reflected in our findings [20].

The relatively higher sensitivity and specificity reported in our study may also reflect greater diagnostic accuracy of PoCUS in patients presenting with acute kidney injury. A recent study evaluating the diagnostic accuracy of PoCUS in patients with acute kidney injury reported a sensitivity of 90% and a specificity of 100% for the detection of hydronephrosis, using departmental ultrasound as the reference standard [25]. Though we report a similar sensitivity to their group, our specificity is lower. This reflects our high false positive rate. However, of the 19 false positive kidney scans included in our analysis, 14 went on to have decompression with Foley insertion. This likely led to an underestimation of overall specificity. This hypothesis is supported by our sensitivity analysis which shows that specificity increases to 89% when all patients with Foley insertion are excluded.

The two main strengths of our study are the use of ICD-10 coding and Kidney Disease Improving Global Outcomes acute kidney injury criteria to capture a large cohort of potentially eligible patients and the broad range of PoCUS providers included. Study limitations include the retrospective, single centre nature of our review and biases. First, our relatively high prevalence of hydronephrosis likely represents a selection bias, in that providers may have been more likely to perform POCUS in patients with a higher pretest probability for obstruction. Additionally, spectrum bias may have contributed to determine a higher accuracy for PoCUS considering that providers may be more likely to record results when they are confident of their findings. Excluding indeterminate tests (*N* = 2) may also have contributed to a higher diagnostic accuracy. Also, considering we used two gold standards, our study may have been subject to differential verification bias. However, this was the most pragmatic approach as both tests are routinely used in the evaluation of patients with AKI. Finally, we acknowledge the potential for change in the state of hydronephrosis within 48 h, which may underestimate accuracy, though median time from PoCUS to computed tomography or radiology ultrasound was 2 h 54 m and 11 h 38 m, respectively. This limitation was also partially addressed through our sensitivity analysis where we excluded all patients with Foley insertion. Considering that documentation of the timing of Foley insertion in relation to imaging was limited, we elected to exclude all patients who had had a Foley inserted in the ED.

## Conclusion

Kidney PoCUS was found to have a sensitivity of 85% and a specificity of 78% for detecting hydronephrosis in patients presenting to our centre with acute kidney injury. For patients who did not undergo the reference standard, we report no missed diagnoses via chart review. PoCUS remains largely underutilized as a diagnostic tool in this patient population at our centre.

### Supplementary Information

**Additional file 1: Table S1.** Comparison of hydronephrosis grading on POCUS and reference standard. **Table S2.** Clinical and technical information of false negative scans.

## Data Availability

The datasets used and/or analysed during the current study are available from the corresponding author on reasonable request.

## Abbreviations

AKI: Acute kidney injury
ED: Emergency department
EM: Emergency medicine
ICD-10: International classification of diseases, tenth revision
LR(+/−): Likelihood ratio (positive/negative)
PoCUS: Point-of-care ultrasound
QI: Quality improvement
ULN: Upper limit of normal
UTI: Urinary tract infection

## Footnotes

1 In the interest of the word limit: “Point of care ultrasound”, “Point-of-care ultrasound”, “Point-of-care-ultrasound”, “Bedside ultrasound”, “Bedside US”, “EMUS”, “EM ultrasound”, “Beside ultrasonography”, “Focused ultrasound”, “FAST”, “RUSH”, “US at bedside”.

## References

[1] Susantitaphong P, Cruz DN, Cerda J (2013). World incidence of AKI: a meta-analysis. Clin J Am Soc Nephrol.

[2] Xue JL, Daniels F, Star RA (2006). Incidence and mortality of acute renal failure in medicare beneficiaries, 1992 to 2001. JASN.

[3] Wonnacott A, Meran S, Amphlett B, Talabani B, Phillips A (2014). Epidemiology and outcomes in community-acquired versus hospital-acquired AKI. Clin J Am Soc Nephrol.

[4] Stucker F, Ponte B, De la Fuente V (2017). Risk factors for community-acquired acute kidney injury in patients with and without chronic kidney injury and impact of its initial management on prognosis: a prospective observational study. BMC Nephrol.

[5] Liano F, Pascual J (1996). Epidemiology of acute renal failure: a prospective, multicenter, community-based study. Madrid Acute Renal Failure Study Group. Kidney Int.

[6] Podoll A, Walther C, Finkel K (2013). Clinical utility of gray scale renal ultrasound in acute kidney injury. BMC Nephrol.

[7] D'Silva K, Dahm P, Wong C (2014). Does this man with lower urinary tract symptoms have bladder outlet obstruction? The rational clinical examination: a systematic review. JAMA.

[8] Kellum JA, Lameire N, Aspelin P (2012). Kidney disease: improving global outcomes (KDIGO) acute kidney injury work group. KDIGO clinical practice guideline for acute kidney injury. Kidney Int Suppl.

[9] Faubel S (2014). Renal relevant radiology: introduction. Clin J Am Soc Nephrol.

[10] Weile J, Brix J, Moellekaer AB (2018). Is point-of-care ultrasound disruptive innovation? Formulating why POCUS is different from conventional comprehensive ultrasound. Crit Ultrasound J.

[11] Koratala A, Ronco C, Kazory A (2022). Multi-organ point-of-care ultrasound in acute kidney injury. Blood Purific.

[12] Herbst MK, Rosenberg G, Daniels B (2014). Effect of provider experience on clinician-performed ultrasonography for hydronephrosis in patients with suspected renal colic. Ann Emerg Med.

[13] Gaspari RJ, Horst K (2005). Emergency ultrasound and urinalysis in the evaluation of flank pain. Acad Emerg Med.

[14] Watkins S, Bowra J, Sharma P, Holdgate A, Giles A, Campbell L (2007). Validation of emergency physician ultrasound in diagnosing hydronephrosis in ureteric colic. Emerg Med Aust.

[15] Torres-Macho J, Antón-Santos JM, García-Gutierrez I (2012). Initial accuracy of bedside ultrasound performed by emergency physicians for multiple indications after a short training period. Am J Emerg Med.

[16] Leo MM, Langlois BK, Pare JR (2017). Ultrasound vs. computed tomography for severity of hydronephrosis and its importance in renal colic. West J Emerg Med.

[17] Pathan SA, Mitra B, Mirza S (2018). Emergency physician interpretation of point-of-care ultrasound for identifying and grading of hydronephrosis in renal colic compared with consensus interpretation by emergency radiologists. Acad Emerg Med.

[18] Sibley S, Roth N, Scott C (2020). Point-of-care ultrasound for the detection of hydronephrosis in emergency department patients with suspected renal colic. Ultrasound J.

[19] Riddell J, Case A, Wopat R (2014). Sensitivity of emergency bedside ultrasound to detect hydronephrosis in patients with computed tomography-proven stones. West J Emerg Med.

[20] Smith-Bindman R, Aubin C, Bailitz J (2014). Ultrasonography versus computed tomography for suspected nephrolithiasis. N Engl J Med.

[21] Wong C, Teitge B, Ross M, Young P, Robertson HL, Lang E (2018). The accuracy and prognostic value of point-of-care ultrasound for nephrolithiasis in the emergency department: a systematic review and meta-analysis. Acad Emerg Med.

[22] Ultrasound Guidelines (2017). Emergency, point-of-care and clinical ultrasound guidelines in medicine. Ann Emerg Med.

[23] Mayo PH, Beaulieu Y, Doelken P (2009). American College of Chest Physicians/La Société de Réanimation de Langue Française statement on competence in critical care ultrasonography. Chest.

[24] Soni NJ, Schnobrich D, Mathews BK (2019). Point-of-care ultrasound for hospitalists: a position statement of the society of hospital medicine. J Hosp Med.

[25] Nepal S, Dachsel M, Smallwood N (2020). Point-of-care ultrasound rapidly and reliably diagnoses renal tract obstruction in patients admitted with acute kidney injury. Clin Med (Lond).

[26] Javaudin F, Mounier F, Pes P (2017). Evaluation of a short formation on the performance of point-of-care renal ultrasound performed by physicians without previous ultrasound skills: prospective observational study. Crit Ultras J.

[27] Kidney Disease: Improving Global Outcomes (KDIGO) Acute Kidney Injury Work Group (2012) KDIGO clinical practice guideline for acute kidney injury. Kidney Int 2(1):1

[28] Coursey CA, Casalino DD, Remer EM (2012). ACR appropriateness criteria^®^ acute onset flank pain–suspicion of stone disease. Ultrasound Q.

