# Supplementary material for: Test characteristics of point-of-care ultrasonography in patients with acute kidney injury
Source: Ultrasound J. 2024 Feb 22;16:15. doi: 10.1186/s13089-023-00352-3 (PMC10884383; doi:10.1186/s13089-023-00352-3)
Supplement: Supplementary file 1 — Additional file 1: Table S1. Comparison of hydronephrosis grading on POCUS and reference standard. Table S2. Clinical and technical information of false negative scans. [file 13089_2023_352_MOESM1_ESM.docx]

**Table S1:** Comparison of hydronephrosis grading on POCUS and reference standard

| Reference standard grade | | | | | |
| --- | --- | --- | --- | --- | --- |
| POCUS grade | None | Mild | Moderate | Moderate to severe | Severe |
| No hydronephrosis | 65 | 2 | 3 | 1 | 0 |
| Mild | 2 | 6 | 4 | 0 | 0 |
| Moderate | 2 | 0 | 5 | 0 | 0 |
| Moderate to severe | 2 | 0 | 1 | 0 | 0 |
| Severe | 4 | 1 | 1 | 0 | 3 |

**Table S2**: Clinical and technical information of false negative scans

|  | Reference standard grade | POCUS provider | M/F | Pertinent PMHx | Presenting complaint | ED primary diagnosis | POCUS Technical factors |
| --- | --- | --- | --- | --- | --- | --- | --- |
| Patient 1  (1 FN) | Mild | EM attending credentialed | F | Recurrent urinary tract infection  Limited functional status – from nursing home | Nausea, vomiting, dysuria and back pain along with an altered mental status | Urosepsis | Documented as very difficult |
| Patient 2  (1 FN) | Moderate | EM attending, credentialed | M | Dementia, rectal malignancy, Limited functional status – from nursing home | Fall and hypotensive | Sepsis | Not documented  Archived images are adequate |
| Patient 3  (1 FN) | Mild | EM attending, credentialed | M | Hydronephrosis with nephrostomy tube, BPH, bladder cancer | Decreased urine output | Renal failure | Not documented  Archived images are poor quality |
| Patient 4  (2 FN) | Moderate to severe | EM resident, credentialed | M | History of bladder cancer and right urothelial cancer | Malaise and weakness with hypotension | Acute kidney injury | Not documented  Archived images are poor quality |
| Patient 5  (1 FN) | Moderate | EM attending, credentialed | M | Obesity (BMI 41)  Remote nephrolithiasis | Dark urine and inability to urinate | Acute kidney injury | Not documented  No images archived |
